# Evaluation of different models of intrusive force application and temporary anchorage device (TAD) placement in total arch intrusion using clear aligners; a finite element analysis

**DOI:** 10.1186/s12903-023-03465-2

**Published:** 2023-10-10

**Authors:** Allahyar Geramy, Soroush Ebrahimi

**Affiliations:** Orthodontics Department, School of Dentistry, Tehran University of Medical Sciences, Tehran, Iran

**Keywords:** Finite element analysis, Total Arch Intrusion, Clear Aligner Therapy, Temporary Anchorage Devices

## Abstract

**Introduction:**

Present study aims to evaluate different models of total arch intrusion using clear aligners in a finite element setup, which might be helpful in gummy smile patients who seek this treatment modality.

**Methods:**

Four patterns of intrusive forces were applied on each side of the upper arch aligner model: (1) Distal to the lateral incisors (facial − 80 g) and distal to the first molar (palatal − 150 g). (2) Distal to the lateral incisors (facial − 80 g) and distal to the first molars (facial − 80 g and palatal − 80 g). (3) Distal to the canines (facial − 80 g) and distal to the first molars (facial − 150 g). (4) Distal to the lateral incisors (facial − 80 g) and mesial to the first molars (facial − 150 g). Vertical and horizontal movements of the teeth were measured.

**Results:**

Extrusion movements were solely detected at buccal cusps of the first and second molars in the first model. Palatal movements of posterior teeth were detected in this model. Model II showed a homogeneous intrusion in anterior and posterior teeth and the amount of palatal movements of posterior teeth was reduced compared to model I. In contrast to Model IV, Model III had more intrusion in the posterior compared to anterior teeth. Facial movements of posterior teeth were detected in the third and fourth models. Incisor teeth showed facial movements among all of the models except for the lateral incisor in the third model.

**Conclusions:**

Each model of force application, causes different outcomes and side effects which is beneficial in certain clinical situations.

## Introduction

With recent advances in orthodontic technologies, a growing number of patients, including gummy smilers, are demanding “invisible” or esthetic treatments like clear aligner therapy. Treatment of patients with excessive gingival display (more than 3 to 4 mm) with or without anterior open bite, depends on various factors. The etiologies include short or hyperactive upper lip, gingival hyperplasia, altered passive eruption and overeruption of anterior and/or posterior dentoalveolar components [1, 2].

In cases of short upper lip, surgical lengthening of the lip is the ideal treatment [3–5] and in hyperactive lips, reduction of the lips mobility is achievable through injection of Botulinum toxin [6]. If gingival hyperplasia is the culprit, gingivectomy or crown lengthening is the treatment of choice [7].

In patients with overeruption of anterior teeth, orthodontic mechanics to intrude incisors such as intruding archwires or Temporary Anchorage Devices (TADs) are helpful [8–10]. In the past years, orthognathic surgery was the only treatment modality for vertical maxillary excess, but by using Temporary Anchorage Devices, effective intrusion of incisors and molars is possible [11–14]. Relatively more intrusion of posterior than anterior teeth, is recommended when anterior open bite is concurrent with excessive gingival display. The opposite is helpful in gummy smilers with deep overbite; more intrusion of anterior teeth relative to posterior teeth, aids in deepbite correction.

Although many articles have addressed using TADs and fixed orthodontic appliances for intrusion [15, 16], only a few case reports have evaluated using TADs and clear aligners for this purpose. Variant locations for TADs placement could lead to different lines of force application and various side effects on the teeth, including palatal or facial crown inclinations. Also, some models of TAD placement could be more efficient in posterior and/or anterior intrusion. No study was found to evaluate the outcomes and side effects of various TAD placement models for total arch intrusion by using clear aligners.

Thus, the aim of present finite element analysis was to compare the results of different models for TAD placement in total arch intrusion using clear aligners.

## Materials and methods

Inspired by an investigation on fixed orthodontic appliance, models were designed in SolidWorks version 2019 (SolidWorks V2019, Dassault Systems, Paris, France) to mimic the condition when forces are applied to upper arch clear aligner for total arch intrusion. These models of force application were designed identical to a similar study on fixed orthodontic appliances to facilitate comparison between these appliances and clear aligners [15].

A model of maxillae, upper dental arch and their periodontal ligaments, spongy and cortical bone and an aligner were designed. The teeth were designed according to Ash’s dental anatomy with a 0.25 mm thickness of periodontal ligament. The differences between models were in the site of intrusive force application:

Model I: Facial intrusive forces were applied at the mesial region of the canines (80 g) on each side and palatal intrusive forces were applied to the aligner at the distal region of the first molars (150 g) on each side.

Model II: Facial intrusive forces were applied at the mesial region of canines (80 g) on each side and also at the distal area of the first molars (80 g) on each side and palatal intrusive forces were applied to the aligner at the distal region of the first molars (80 g) on each side.

Model III: Facial intrusive forces were applied distal to the canines (80 g) on each side and also distal to the first molars (150 g) on each side.

Model IV: Facial intrusive forces were applied in the mesial areas of the canines (80 g) on each side and also at the mesial areas of the first molars (150 g) on each side.

In each models, TADs were placed according to the point of force applications. For facial forces, if the forces were applied at the mesial or distal regions of the canines, the facial mini-screws were placed between the lateral incisors and canines or between the first premolars and canines at the mucogingival junction level, respectively. Similarly, if the forces were applied at the mesial or distal regions of the first molars, the facial mini-screws were placed between the second premolars and first molars or between the first and second molars at the mucogingival junction level, respectively. In the first and second models, the palatal mini-screws were placed at the mid palatal suture in the area between the first and second molars.

**Fig. 1:**
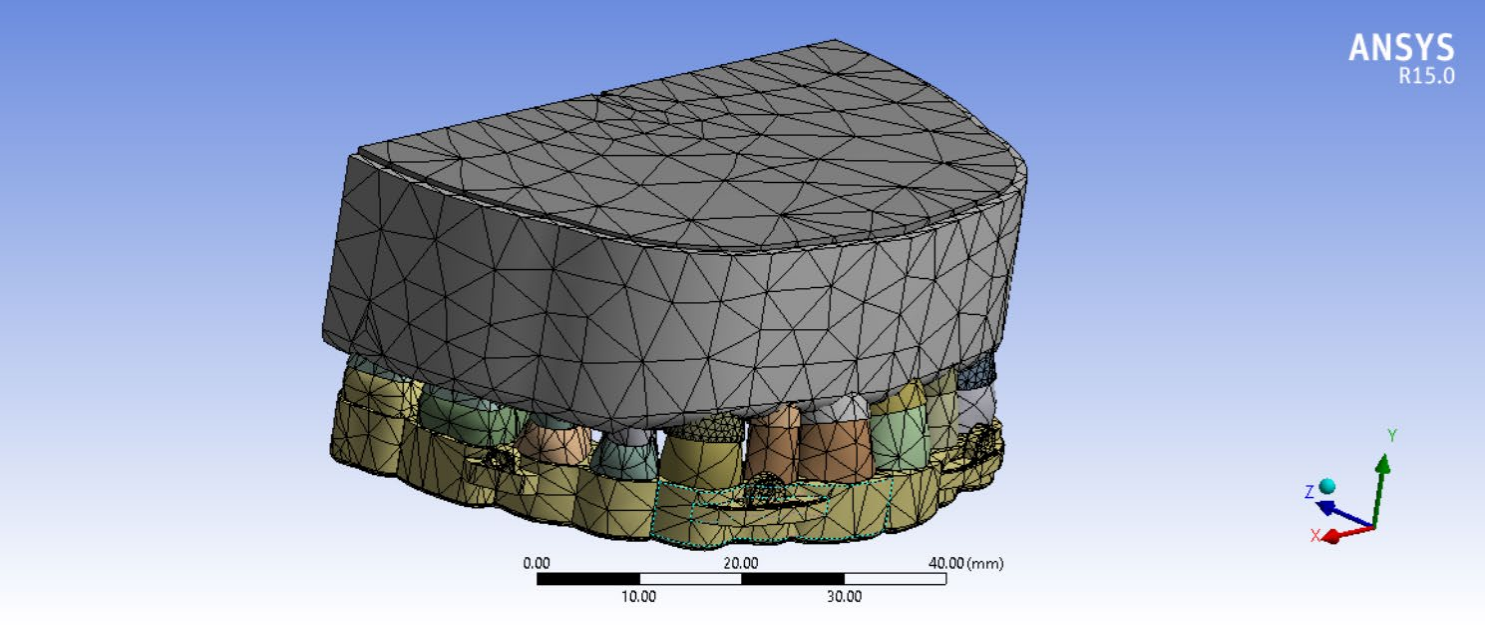
Meshed 3-D computer model of upper dental arch and an aligner. Note the designed hooks on the aligner at the mesial region of canines and first molar (Model IV) where vertical intrusive forces were applied

The models were transferred to ANSYS Workbench V15.0 (ANSYS Inc., Canonsburg, Pennsylvania USA). Mechanical properties of the materials were then employed and the models were meshed (Table 1; Fig. 1).

**Table 1:** The mechanical properties of the materials used in the models

	Young’s Modulus	Poisson’s Ratio
Spongy Bone	13,400	0.38
Cortical Bone	34,000	0.26
Tooth	20,300	0.26
Periodontal Ligament	0.667	0.49
Titanium (Miniscrew)	96,000	0.36
Clear Aligner (Duran)	2227	0.36

The mechanical properties of aligner used in the present study are compatible with the material properties of Duran aligner with 1 mm of thickness [17, 18]. The intrusive forces were applied vertically to the hooks designed into the aligners with no horizontal component.

The models were solved to obtain the vertical (intrusion/extrusion) and the horizontal (palatal/facial) movements of the teeth. Movements of the teeth were evaluated at the midpoint of the incisal edges of central and lateral incisors, at the cusp tips of canines and molars (buccal and palatal cusps for molars) and the central grooves of the premolars.

## Results

### Vertical displacements

The vertical effects of different models of intrusive force application are summarized in Table 2.

**Table 2:** The vertical displacements (in millimeters) of the teeth in different models. Positive values indicate intrusion and negative values show extrusion

Tooth/Point	Model I	Model II	Model III	Model IV
Central Incisor	1.06 × 10^− 3^	8.13 × 10^− 4^	1.62 × 10^− 4^	8.39 × 10^− 4^
Lateral Incisor	9.02 × 10^− 4^	7.78 × 10^− 4^	3.61 × 10^− 4^	9.74 × 10^− 4^
Canine	6.48 × 10^− 4^	6.65 × 10^− 4^	6.03 × 10^− 4^	1.06 × 10^− 3^
First Premolar	4.48 × 10^− 4^	5.3 × 10^− 4^	6.27 × 10^− 4^	9.18 × 10^− 4^
Second Premolar	4.1 × 10^− 4^	5.18 × 10^− 4^	6.41 × 10^− 4^	8.41 × 10^− 4^
MB First Molar	-1.81 × 10^− 4^	4.38 × 10^− 4^	1.11 × 10^− 3^	9.82 × 10^− 4^
DB First Molar	-4.06 × 10^− 5^	5.17 × 10^− 4^	1.1 × 10^− 3^	7.16 × 10^− 4^
P First Molar	8.58 × 10^− 4^	7.19 × 10^− 4^	1.01 × 10^− 3^	3.95 × 10^− 4^
MB Second Molar	-1.29 × 10^− 4^	5.61 × 10^− 4^	1.23 × 10^− 3^	4.59 × 10^− 4^
DB Second Molar	-2.09 × 10^− 5^	5.66 × 10^− 4^	1.14 × 10^− 3^	2.71 × 10^− 4^
P Second Molar	1.06 × 10^− 3^	8.45 × 10^− 4^	1.28 × 10^− 3^	1.91 × 10^− 5^

MB, Mesio-buccal cusp; DB, Disto-buccal cusp; P, Palatal cusp.

**Fig. 2:**
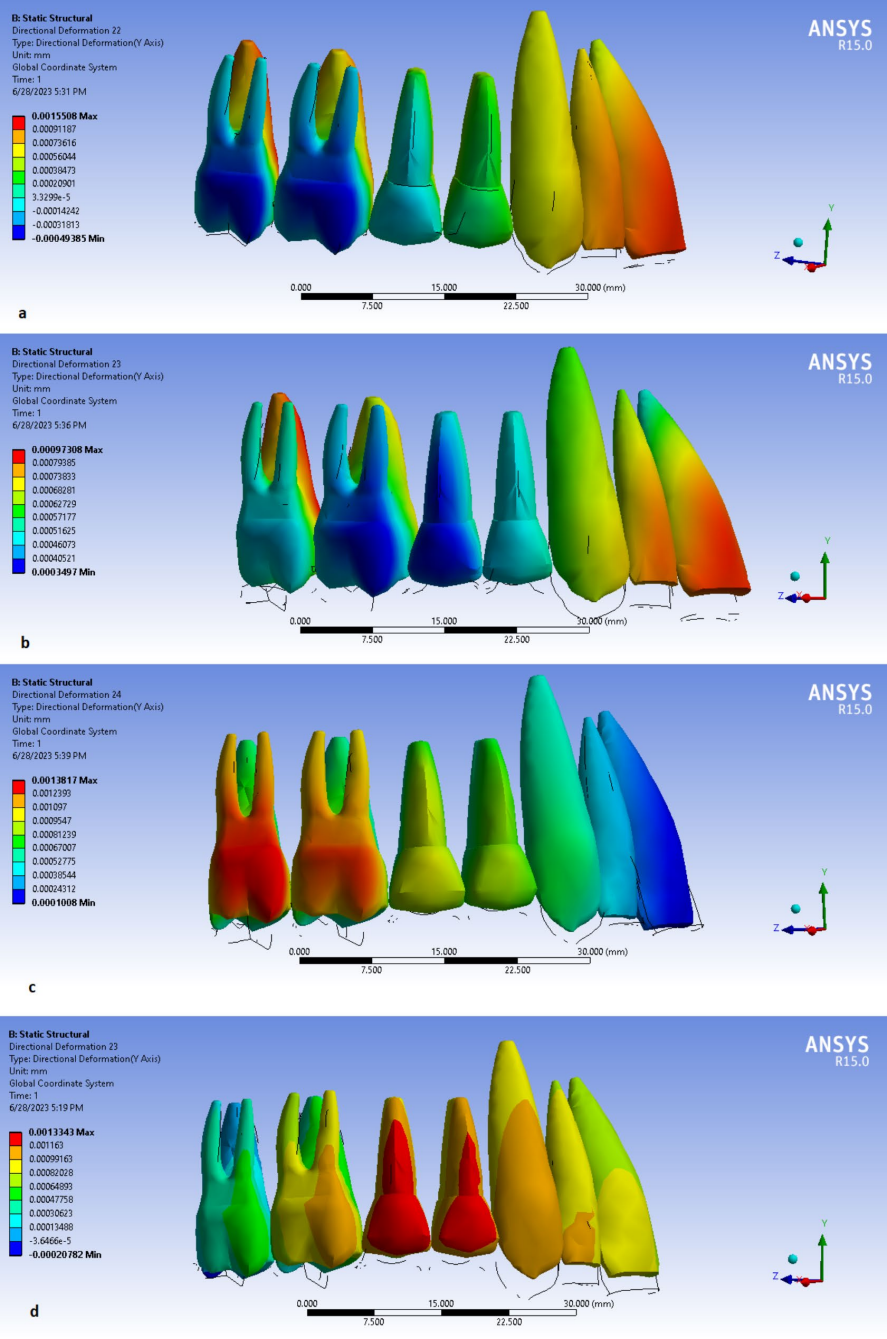
The vertical displacements of designed teeth in different models. **a**, Model I; **b**, Model II; **c**, Model III and **d**, Model IV.

*Model I.* The intrusion of anterior teeth and premolars was observed. In the molar area, intrusion of palatal cusps and extrusion of buccal cusps was noted. Relatively more intrusion in incisors was detected than in canines and premolars. Also, palatal cusps of molars had more intrusion than premolars and canines but were almost similar to incisors (Fig. 2a).

*Model II.* The intrusion of all points was noted. The intrusion of incisors was more than canines and premolars and almost as much as the palatal cusps of molar teeth. Relatively more intrusion was observed in palatal cusps of molar teeth than in buccal cusps (Fig. 2b).

*Model III.* A gradient of intrusion was detected. Posterior points had relatively more intrusion than anterior points. Buccal and palatal cusps of molars had almost similar amounts of intrusion (Fig. 2c).

*Model IV.* The first molars had more intrusion than the second molars and the intrusion of palatal cusps was less than buccal cusps. Canines showed more intrusion relative to incisors and premolars (Fig. 2d).

### Anterior/posterior or medial/lateral displacements

Table 3 shows the facial or palatal coronal movements of the teeth in different models.

**Table 3:** The horizontal displacements (in millimeters) of the teeth in different models. Positive values indicate facial movements and negative values show palatal displacements

Tooth/Point	Model I	Model II	Model III	Model IV
Central Incisor	8.01 × 10^− 4^	3.45 × 10^− 4^	1.27 × 10^− 5^	3.76 × 10^− 5^
Lateral Incisor	5.98 × 10^− 4^	2.86 × 10^− 4^	-1.66 × 10^− 5^	2.27 × 10^− 4^
First Premolar	-6.18 × 10^− 4^	-1.89 × 10^− 4^	4.24 × 10^− 4^	3.68 × 10^− 4^
Second Premolar	-1.11 × 10^− 3^	-3.32 × 10^− 4^	6.38 × 10^− 4^	4.76 × 10^− 4^
MB First Molar	-1.86 × 10^− 3^	-5.34 × 10^− 4^	1.0 × 10^− 3^	7.39 × 10^− 4^
DB First Molar	-2.1 × 10^− 3^	-6.29 × 10^− 4^	1.01 × 10^− 3^	7.15 × 10^− 4^
P First Molar	-2.03 × 10^− 3^	-5.98 × 10^− 4^	1.01 × 10^− 3^	7.14 × 10^− 4^
MB Second Molar	-2.65 × 10^− 3^	-7.62 × 10^− 4^	1.27 × 10^− 3^	7.7 × 10^− 4^
DB Second Molar	-2.7 × 10^− 3^	-7.61 × 10^− 4^	1.29 × 10^− 3^	7.14 × 10^− 4^
P Second Molar	-2.67 × 10^− 3^	-7.56 × 10^− 4^	1.28 × 10^− 3^	7.21 × 10^− 4^

MB, Mesio-buccal cusp; DB, Disto-buccal cusp; P, Palatal cusp.

**Fig. 3:**
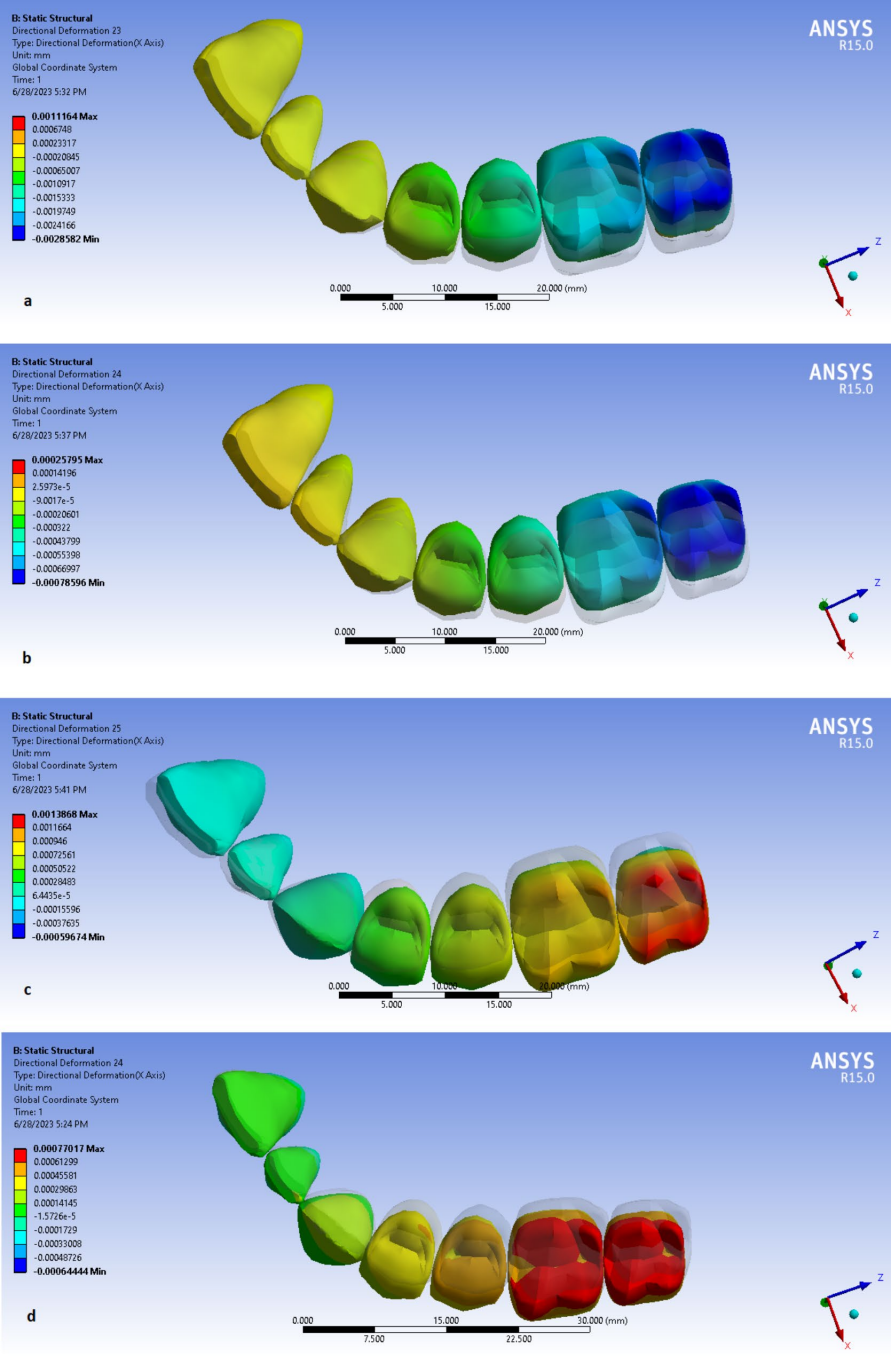
The lateral displacements of designed teeth in different models. **a**, Model I; **b**, Model II; **c**, Model III and **d**, Model IV.

*Model I.* The intrusive forces resulted in the facial movements of the incisors and the palatal movements of all the posterior teeth. More posterior teeth had more palatal movements (Fig. 3a).

*Model II.* This model had similar results to the first model except that the amounts of palatal or facial movements were less than the previous model (Fig. 3b).

*Model III.* All of the points except the lateral incisor, had facial movements. More posterior teeth had more movements than anterior teeth (Fig. 3c).

*Model IV.* The facial movements of all the points were detected. Molars had more facial movements than premolars (Fig. 3d).

## Discussion

The purpose of the present study was to evaluate four models of TAD placement and intrusive force application for total arch intrusion by clear aligners. Intrusive forces were applied to the virtual designs of upper arch clear aligners and the models were analyzed to calculate vertical and horizontal displacements of the teeth. The current finite element analysis could be a basis for future clinical experiments.

A review conducted by Rossini et al. suggested that clear aligner therapy might be effective in performing anterior intrusion for the treatment of mild deep overbite discrepancies [19]. Also, there is no significant difference in the accuracy of the actual and predicted intrusion of anterior teeth using aligners [20]. The accuracy of the maxillary teeth intrusion using clear aligners ranges from 33.4 to 53.3% and the intrusion of posterior teeth, the same as anterior teeth, is achievable using aligners [21].

In general, successful intrusion of the teeth was observed in different models of intrusive force application; the only exception was buccal cusps of the molar teeth in the first model which showed extrusion. This problem was resolved by adding another intrusive force to the facial side and reducing the palatal force (Model II).

The first and second models caused palatal crown flaring of molars and palatal displacement of premolars and also more intrusion in the palatal than buccal cusps of molars. This might be helpful in patients when over-eruption of posterior teeth is concurrent with a posterior crossbite. Palatal expansion for the treatment of posterior crossbite causes buccal flaring of posterior teeth and possible overhanging of palatal cusps especially in post-adolescent patients [22–24]. These models prevent over buccal flaring of posterior teeth in these patients and the second model seems to be a better option since unlike the first model, it causes intrusion of buccal cusps in molars.

By comparing the results of the first and second models, the consequences of adding two intrusive forces to the facial sides of molar teeth and reducing the palatal forces are revealed. These facial forces prevent the extrusion of buccal cusps of molars and reduce palatal displacements of posterior teeth and facial displacements of central and lateral incisors. Also, the amount of intrusion in the central and lateral incisors and palatal cusps of molars is reduced, but premolar intrusion is increased.

Compared to the first model, the second model has a more balanced form of total arch intrusion; meaning that the amounts of intrusion in different teeth (i.e. incisors, canines, premolars and molars) are more homogeneous while in the first model, more intrusion is detected in the incisors and palatal cusps of molars compared to the canine and premolars.

Intrusive forces in the third model are more distally positioned compared to the fourth model. Placement of mini-screws in more mesial positions had two major effects. First, the intrusion of incisors, canines and premolars was increased and the amount of molar intrusion was decreased. Second, facial displacement of posterior teeth (molars and premolars) is decreased, but there is more facial displacement of incisors in the fourth model (the lateral incisors had palatal displacement in the third model and facial displacement in the fourth model).

Intrusion of maxillary posterior teeth could help in the correction of moderately severe anterior open bites and a decrease in anterior face height [25]. Since the amount of intrusion of the molars is higher than incisors and canines in the third model, it seems to be beneficial in patients with over-eruption of anterior and posterior teeth and anterior open bite. Open bite closure by clear aligner therapy without using the mini-screws is usually achieved through a combination of maxillary and mandibular incisor extrusion and maxillary and mandibular molar intrusion and a slight mandibular auto-rotation [26, 27]. The third model probably has the same clinical effects but it also prevents the extrusion and possible over-display of maxillary incisors.

On the other hand, intrusion of maxillary anterior teeth could lead to the correction of excessive anterior gingival display and deep overbite [28, 29]. Because more intrusion was observed in incisors and canines than the molar teeth in the fourth model, it might be helpful in patients with over-eruption of anterior and posterior teeth and deep overbite.

When comparing all the models, in general, the highest amounts of intrusion in the incisor, canine and premolar areas were noticed in the fourth model, supporting the idea that this model might be advantageous in deep bite patients. On the other hand, the highest amount of molar intrusion is detected in the third model; again suggesting that it is applicable in open bite cases.

The fact that the lowest amounts of intrusion in the incisors and canine areas were detected in the third model, may suggest that this model is useful in patients with good to excessive eruption of anterior teeth. Conversely, the fourth model had the lowest amounts of the molars intrusion, especially in the palatal cusps which might imply that this model is suitable for patients with good to excessive eruption of the molar teeth.

Although finite element analyses could be a basis for understanding of the effects of force application to different models, there are some limitations. The results of these model are not directly applicable to the clinical situations and future clinical studies with similar models of force application are necessary. The reason for these shortcomings is that finite element analyses could not completely simulate oral environment and factors such as biological organisms, muscular functions and saliva might alter clinical outcomes of orthodontic treatments. In addition, these analyses do not reveal the long term results of the treatments. Despite all of these limitation, finite element analyses could reduce sample size and expenses of the future clinical studies.

## Conclusions

Considering all of the limitations, the following conclusions are derived from the present finite element study:

1. Total arch intrusion is achievable using a combination of clear aligners and temporary anchorage devices (TADs).
2. In patients with a posterior crossbite, the first and second models are beneficial to prevent over-hanging of palatal cusps of molars and buccal flaring of posterior teeth. The second model is preferable since it prevents extrusion of buccal cusps and reduces palatal displacements of posterior teeth.
3. Placing the TADs in more posterior regions (like Model III), might help in anterior open bite closure as well as total arch intrusion.
4. Placing the TADs in more anterior regions (like Model IV), might help in deep bite correction as well as total arch intrusion.

## Data Availability

All data generated or analyzed during this study are included in this published article.
