# Correction: Evaluation of different models of intrusive force application and temporary anchorage device (TAD) placement in total arch intrusion using clear aligners; a finite element analysis

**DOI:** 10.1186/s12903-023-03562-2

**Published:** 2023-11-28

**Authors:** Allahyar Geramy, Soroush Ebrahimi

**Affiliations:** Orthodontics Department, School of Dentistry, Tehran University of Medical Sciences, Tehran, Iran

**Correction: BMC Oral Health 23, 740 (2023)**

**https://doi.org/10.1186/s12903-023-03465-2**

In the original version of this article [1], the given and family name of all the authors were incorrectly structured. Instead of Geramy A and Ebrahimi S, the authors name was incorrectly tagged as Allahyar G and Soroush E.

The original article has been corrected.
